# Daily versus intermittent iron supplementation among children with iron deficiency anemia: A meta-analysis

**DOI:** 10.12669/pjms.40.11.10344

**Published:** 2024-12

**Authors:** Sawsan Mohammed Alblewi

**Affiliations:** Sawsan Mohammed Alblewi Pediatrics Department, Faculty of Medicine, University of Tabuk, Saudi Arabia, PO Box 3378 Tabuk 51941, Saudi Arabia

**Keywords:** Iron supplementation, Daily, Intermittent, Children, Iron deficiency anemia

## Abstract

**Objective:**

This meta-analysis aimed to investigate daily versus intermittent iron supplementation among children.

**Methods:**

The author searched the Cochrane Library, PubMed, Medline, and Google Scholar for randomized controlled trials and compared daily versus intermittent iron supplementation. The keywords iron deficiency anemia, iron therapy, daily, twice per week, and once weekly were used. The search was limited to the period January 2012 up to November 2023. The age of participant range from two months to 18 years.

**Result:**

Out of the 735 studies, 540 were eligible after removal of duplication, of them, 28 full texts were screened and only 10 studies were included. Daily iron supplementation was better in improving hemoglobin odd ratio, 0.41, 95 CI, 0.38-0.44, Z=26.53, and p-Value <0.001 than intermittent prescription odd ratio, 0.69, 95 CI, 0.67-0.72, Z=49.98, and P-value < 0.001.

**Conclusion:**

Daily supplementation increased hemoglobin more compared to weekly or twice/week among children with iron deficiency anemia. Further larger studies assessing tolerance and compliance are needed.

## INTRODUCTION

Iron deficiency anemia is the most common cause of anemia in the world. One-third of people worldwide suffer from anemia, with iron deficiency accounting for half of the instances.^1^,^2^ An estimated 273 million children under the age of five were anemic worldwide in 2011, with iron deficiency being the cause of roughly half of those instances.^3^ There have been reports of IDA prevalence in Saudi Arabia ranging from 10% to 60% overall.^4^

The symptoms of iron deficiency anemia in children are feeling exhausted, and weak, experiencing lightheadedness and dizziness, having trouble focusing and remembering things, and performing poorly at school. In addition, to negative immune system effects. In more extreme situations, the requirement to raise cardiac output results in palpitations and dyspnea.^5^ Moreover, iron deficiency anemia stunted children’s psychomotor development.^6^

Diagnosis of iron deficiency anemia depends on a complete blood count to determine the hemoglobin and the mean corpuscular volume; microcytic anemia is highly suspicious with ferritin levels below the normal range confirming the diagnosis.^7^ Management of IDA is by iron supplement either oral or intravenous, the dose of oral iron therapy varies from 3 to 6 mg/kg/day, and the frequency is either daily, twice per week, or weekly to avoid side effects and for a better complaint.^8^

The first three years of life are critical for organ development including the brain, iron stores are important for biochemical reactions and organ development.^9^ Evidence from human and animal studies confirmed that brain iron deficiency occurs before red blood cells.^10^ Importantly, brain iron deficiency in childhood negatively impacts brain function in adult life, poor academic achievement, lower job opportunities, and psychiatric diseases.^11^

Daily iron supplementation is recommended after six months of age depending on the geographical location and the prevalence of iron deficiency.^12^ However, adherence issues and the fact that gastrointestinal cells are renewed every five to six days with poor absorption of irons. Therefore, intermittent iron supplementation was suggested to overcome non-compliance and enhance iron absorption.^13^

Intermittent versus daily iron supplementation was assessed in pregnant women.^14^,^15^ Literature regarding the effects of intermittent versus daily iron supplementation in children is scarce. Therefore, the current review aimed to compare Hemoglobin levels after three months of daily, twice-per-week, and once-weekly oral iron therapy.

## METHODS

### Eligibility Criteria according to Population, Intervention, Comparison, Outcomes and Study PICOS:

This meta-analysis was conducted during November and December 2023, the PRISMA guidelines were strictly followed.

### Inclusion criteria:

We considered whether the investigations were prospective or retrospective cohorts, case-control, cross-sectional, or randomized controlled trials (RCTs). Studies that reported oral iron therapy once a day, twice a week, or weekly were eligible. Randomized controlled trials assessing iron supplementation (supervised and unsupervised) among children were included. Simple random technique was considered for the included studies.

### Exclusion criteria:

Prospective or retrospective cohorts, case-control, cross-sectional, animal research, case reports, case series, protocols without data, and systematic reviews and meta-analyses were not pursued. Patients belonging to specific sub-groups and those with any chronic disease were not included. The study duration of less than four weeks, and studies conducted among adults were not eligible.

### Outcome measures:

Hemoglobin increment after oral iron therapy where is it once daily, twice per week, or once weekly?

### Information sources and search:

We searched the Cochrane Library, PubMed, Medline, and Google Scholar. The search was conducted during November and December 2023 and was limited to the period January 2012 up to November 2023. The search was limited to articles published in the English language. The keywords iron deficiency anemia, iron therapy, daily, twice per week, and once weekly were used. Out of the 735 studies, 540 were eligible after removal of duplication, of them, 28 full texts were screened and only 10 studies were included in the final analysis.

### Data extraction:

**Fig.1:**
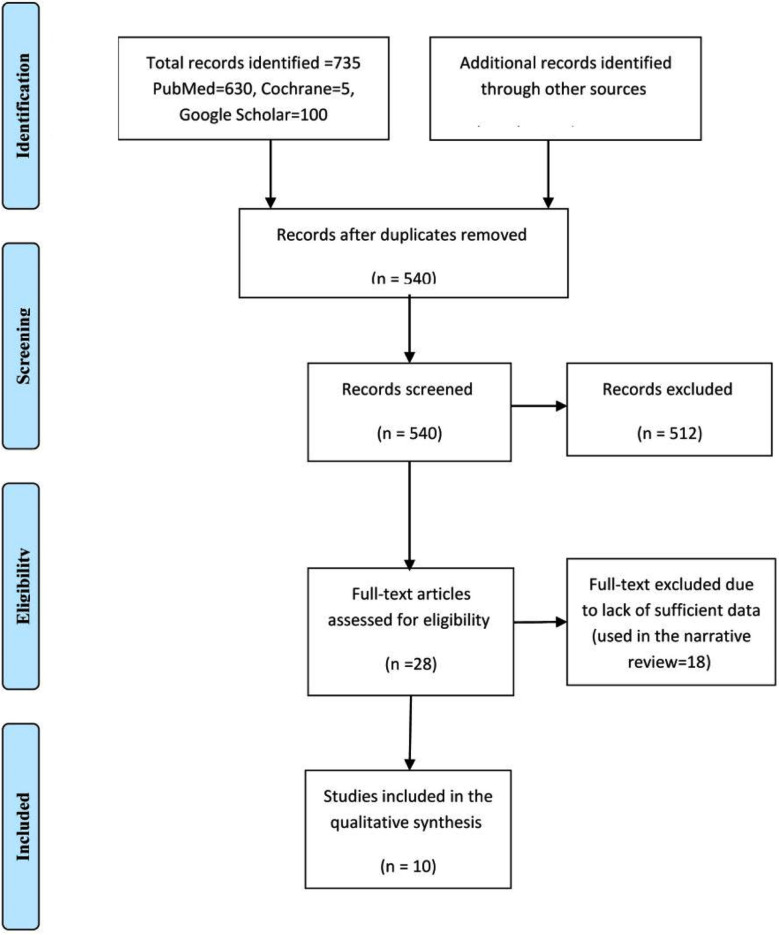
Daily versus weekly iron supplementation (the PRISMA Chart).

An Excel sheet detailing the author’s name, year of publication, number of patients, and the increment of hemoglobin level in various frequencies used daily, twice per week, or once weekly was used. A modified Cochrane Risk of Bias (ROB-2) assessed the quality of the included study.^16^ Fig.1 and Tables-I and II.

**Table-I:** Randomized trials investigating daily versus intermittent iron supplementation among children with iron deficiency anemia.

Author name	Age year	Duration of study	Does daily vs intermittent	Gender girl % daily vs intermittent	Country	Daily g/dL	Twice/week	Weekly
Berger et al. 1997^17^	3.3-8.3	16 weeks	3-4 mg /kg of elemental iron	53.4 vs 48.3	Bolivia	1.86±0.59/58	-	1.52±0.17/58
Faqih et al. 2006^18^	2-6	12 weeks	5 mg/kg ferrous sulfated	38 vs 42.8	Jordon	2.47 ± 0.17/21	2.12 ± 0.18/21	2.18 ± 0.18/21
Hyder et al. 2007^19^	1-2	8 weeks	12.5 mg vs 30 mg elemental iron	47.1 vs 47	Bangladesh	1.61±1.32/70	-	1.23±1.33/66
Kapil et al. 2013^20^	3-5	14 weeks	20-40 vs 40mg iron	-	India	1.5±0.3/226	-	0.6±0.1/110
Khademloo et al. 2009^21^	0.5-2	12 weeks	15 vs 30 drops of iron	50	Iran	0.6±0.17/50	-	0.4±0.33/50
Siddiqui et al. 2004^22^	5-10	8 weeks	200 mg ferrous sulfate	50	Pakistan	2.5±0.3/30	-	2.02±0/30
Sungthong et al. 2002^23^	6 -13	16 weeks	60 mg of elemental iron	50 vs 52.	Thailand	6.5±6.0/39	-	5.7± 6.3/40
Desai et al. 2004^24^ Unsupervised	2-59 m	12 weeks	3-6 mg/kg vs 6-12 mg/kg ferrous sulfate	51 vs 49	USA	0.82±0.2/251	0.47±0.21/271	-
Desai et al. 2004^24^ supervised	2-59 m	12 weeks	3-6 mg/kg vs 6-12 mg/kg ferrous sulfate	46 vs 51	USA	1.15±0.2/261	0.59±0.21/266	-
Gunadi et al. 2009^25^	9-12	4 weeks	5 mg/kg/day elemental iron	54.7 vs 47.2	Indonesia	4.81±0.59/50	2.26±0.02/47	-
Zavaleta et al. 2000^26^	12-18	17 weeks	60 mg ferrous sulfate	100	Peru	1.11±0.9/20	0.68±0.8/18	-

**Table-II:** Risk of bias assessment of the included studies according to Cochrane risk of bias of randomized controlled trials.

Author	Selection bias^1^	Selection bias^2^	detection	Attrition bias	Reporting bias	Other bias
Berger et al.1997^17^	Unknown	Low	Low	Low	Unknown	Low
Faqih et al.2006^18^	Low	Unknown	High	High	High	High
Hyder et al.2007^19^	Low	Low	Unknown	Unknown	Low	Low
Kapil et al.2013^20^	Low	Low	Unknown	Unknown	Low	Unknown
Khademloo et al.2009^21^	Unknown	Unknown	High	Unknown	Unknown	Low
Siddiqui et al.2004^22^	Unknown	Unknown	High	Low	Unknown	Unknown
Sungthong et al.2002^23^	Low	Low	Low	Low	Unknown	Unknown
Desai et al.2004^24^ Unsupervised	Low	Low	High	Low	Unknown	Unknown
Desai et al.2004^24^ supervised	Low	Low	High	Low	Unknown	Unknown
Gunadi et al.2009^25^	Low	Low	High	Unknown	Unknown	Unknown
Zavaleta et al.2000^26^	Low	Low	low	Low	Unknown	Low

### Data analysis:

The data were all continuous and were processed using the RevMan meta-analysis tool (version, 5.4,1, United Kingdom). For the continuous data, a 95% CI of 5% was used. Depending on the degree of heterogeneity, either the fixed effect or the random effect was applied. Sensitivity analysis was done by removing studies with a high risk of bias. The assessment of lateralization was done using Funnel plots. The p-Values <less 0.05 were regarded as significant.

## RESULTS

### Characteristics of the included studies:

We included 10 clinical trials; two were from South America, seven from Asia, and one from USA.

### Findings:

**Fig.2:**
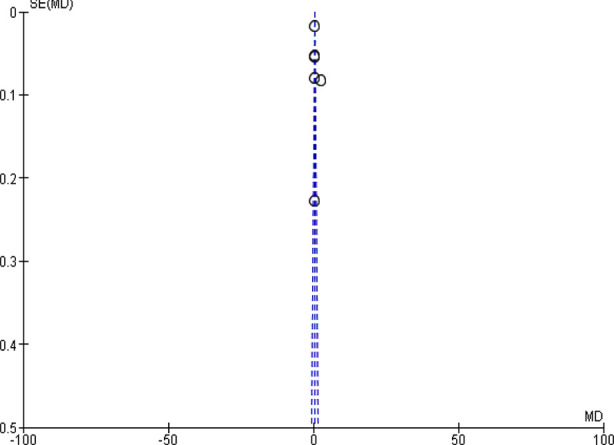
Daily versus weekly iron supplementation in children with iron deficiency anemia (unsupervised).

In this meta-analysis, seven unsupervised studies including 1073 children were pooled.^17^-^23^ Daily iron supplementation was better than intermittent in improving hemoglobin, odd ratio, 0.41, 95 CI, 0.38-0.44, Z=26.53, and *p*-value <0.001. The Chi-square=686.14. A significant heterogeneity was found, I^2^=99%, *p*-value for heterogeneity <0.001, and the standard difference = 5. Fig.2.

**Fig.3:**
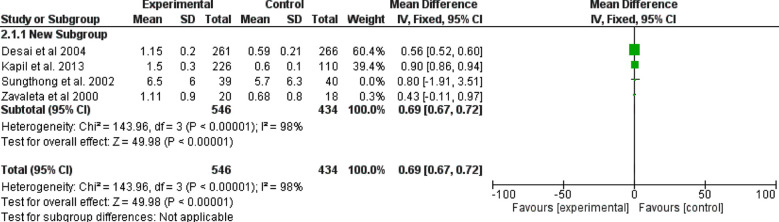
Daily versus intermittent iron supplementations (supervised).

Four studies (980 children included) assessed the difference between daily and intermittent supervised iron supplementation.^18^,^24^-^26^ They showed that daily iron supplementation was better, with odd ratio, 0.69, 95 CI, 0.67-0.72, Z=49.98 and *p-value* ≤ 0.001. The Chi-square=143.96. A significant heterogeneity was found, I^2^=98%, *p*-value for heterogeneity <0.001. Fig.3.


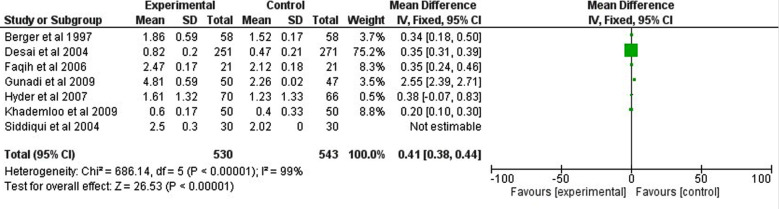


## DISCUSSION

The current findings showed that daily iron supplementation was superior to twice/week, and weekly iron supplementation, odd ratio, 0.41, 95 CI, 0.38-0.44, Z=2.15 and *p*-value < 0.001, A sub-analysis including only supervised iron supplementation confirmed the same, odd ratio, 0.69, 95 CI, 0.67-0.72, Z=49.98 and *p*-value < 0.001. Previous meta-analyses conducted in children showed the benefit of daily iron supplementation on hemoglobin levels with limited evidence on adherence, side effects, and clinical outcomes.^27^-^29^

The available studies assessed the effects of daily iron supplementation, however, meta-analyses comparing daily, and intermittent iron supplementation are scarce. De-Regil et al^30^ compared daily iron supplementation versus placebo and intermittent supplementations. The study included children with and without anemia. Importantly, the authors included only six studies comparing daily versus intermittent intake. In addition, the study was published in the year 2011 and new trials were published.^20^,^31^ therefore, an update is justifiable.

A recent meta-analysis included 129 trials and showed similar effects of frequent and intermittent iron supplementation in contradiction to the current findings.^32^ However, the authors searched the literature up to 2020 and the significant heterogeneity limited their conclusions. In addition, the author included folate supplementation, indicators of infection, and child development. The contradicting findings might have been explained by the fact that Andersen compared 1-2/week versus 3-7/week a frequency that is not so different.

The World Health Organization recommended supplementation of iron in regions with a high prevalence of iron deficiency anemia.^33^ However, gastrointestinal side effects of iron are a major challenge.^33^ Our findings supported the previous finding that daily iron supplementation was better than infrequent iron therapy. However, intermittent iron supplementation is an effective alternative. There is existing evidence that gastrointestinal cells renewed every six days.

Therefore, daily, or twice weekly supplementation bears the advantage of more tolerance and lower side effects.^34^ New iron formulations with few gastrointestinal side effects might solve the problem of intolerance.^35^ An interesting finding of reduced iron transportation from the gastrointestinal tract to the blood following larger doses of iron, pointed to the importance of doses of iron prescription.^36^ This meta-analysis included an up-to-date and larger number of randomized trials and showed the superiority of daily iron supplementation compared to intermittent schedules. In addition, the current findings were different from the previous studies that showed similar efficacy of daily and intermittent prescriptions.^31^,^32^

International organizations stated that supervised iron supplementation could be a better strategy than unsupervised recommendations in reducing anemia among children. In addition, more research is suggested to compare biweekly supervised iron supplementation with the loose strategy of recommending 100 days/year of continuous dosing.^37^ In the current meta-analysis, we assessed both supervised and unsupervised iron supplementation and both were shown to be effective.

Although iron supplementation was effective in the reduction of anemia. However, neurodevelopmental issues, interaction with other trace elements, increased incidence of infection, and gut microbiota disturbance are major concerns^38^ excess iron competes with trace elements absorption and masks zinc and copper deficiency.^39^ Importantly, iron supplementation for prevention and in areas of low iron deficiency might lead to cell death, organ damage, and mortality. Iron overload leads to high oxygen free radicals and oxidative stress inducing oxidation of lipids, proteins, and DNA leading to cell death (ferroptosis).^40^ In addition, iron deposits in the gastrointestinal tract lead to gut dysbiosis with the abundance of Escherichia/Shigella, and Clostridium, and reduction of Bifidobacterium infantis. The above results in microbiota shift and infections.^41^,^42^

### Limitation

It includes the short duration of the trials included and the high heterogeneity observed.

## CONCLUSION

Daily iron supplementation is better than the weekly and twice/week approach among children with iron deficiency anemia. The results remain robot after assessing only supervised iron therapy.
